# Maternal Serum SIRT1 Concentrations in Intrahepatic Cholestasis of Pregnancy: Limited Diagnostic Utility in a Prospective Case—Control Study

**DOI:** 10.3390/diagnostics16121834

**Published:** 2026-06-13

**Authors:** Dinçer Sümer, Ahmet Arif Filiz, Özgür Volkan Akbulut, Figen Günday, Gülten Çirkin Tekeş, Kutlay Bülbül, Demet Sümer, Belgin Savran Üçok, Kadriye Yakut Yücel

**Affiliations:** 1 Department of Perinatology, University of Health Sciences, Etlik City Hospital, Etlik Şehir Hastanesi Varlık Mahallesi Halil Sezai Erkut Cd. No: 5, Yenimahalle, 06170 Ankara, Türkiye; ahmetarif_filiz@hotmail.com (A.A.F.); akbulutvolkan@yahoo.com (Ö.V.A.); figengunday1979@gmail.com (F.G.); gultencir@hotmail.com (G.Ç.T.); yakutkadriye@hotmail.com (K.Y.Y.); 2 Department of Obstetrics and Gynecology, University of Health Sciences, Etlik City Hospital, Varlık Mahallesi Halil Sezai Erkut Cd. No: 5, Yenimahalle, 06170 Ankara, Türkiye; kutlayblbl@gmail.com (K.B.);; 3 Department of General Surgery, Division of Surgical Oncology, University of Health Sciences, Başakşehir Çam and Sakura City Hospital, Başakşehir Mahallesi G-434 Caddesi No: 2L, Başakşehir, 34480 Istanbul, Türkiye; demetsumer@gmail.com

**Keywords:** intrahepatic cholestasis of pregnancy, SIRT1, inflammation, bile acids, pregnancy

## Abstract

**Objective**: To investigate maternal serum silent information regulator-2 protein 1 (SIRT1) levels in pregnancies complicated by intrahepatic cholestasis of pregnancy (ICP) and evaluate their diagnostic performance. **Methods**: This prospective case–control study included 44 pregnant women with ICP and 44 healthy pregnant controls matched according to gestational age at blood sampling and maternal body mass index. Maternal serum SIRT1 concentrations were measured using enzyme-linked immunosorbent assay (ELISA). Clinical, laboratory, and obstetric outcomes were compared between groups. Correlation, receiver operating characteristic (ROC) curve, and exploratory multivariable logistic regression analyses were performed. **Results**: Maternal serum SIRT1 levels were significantly lower in the ICP group compared with controls [1.06 (1.05) ng/mL vs. 1.54 (1.74) ng/mL, *p* = 0.005]. ROC analysis demonstrated modest discriminative performance of maternal serum SIRT1 alone for identifying ICP (AUC: 0.674, 95% CI: 0.559–0.788, *p* = 0.005). A SIRT1 cut-off value of ≤1.28 ng/mL yielded 63.6% sensitivity and 60.5% specificity. In contrast, ALT alone showed excellent discriminative performance (AUC: 0.927, 95% CI: 0.860–0.995, *p* < 0.001). Combined ROC analyses demonstrated further improvement with the ALT + albumin model (AUC: 0.962, 95% CI: 0.925–0.999), whereas addition of SIRT1 resulted in only a minimal incremental increase in AUC to 0.966 (95% CI: 0.933–0.998). Maternal serum SIRT1 concentrations were not independently associated with ICP after adjustment for laboratory parameters. **Conclusions**: Although maternal serum SIRT1 levels were significantly reduced in pregnancies complicated by ICP, their diagnostic performance was modest and provided minimal incremental value beyond conventional biochemical markers. Nevertheless, reduced maternal serum SIRT1 concentrations may support the involvement of inflammatory and oxidative stress-related pathways in ICP pathophysiology and warrant further mechanistic investigation.

## 1. Introduction

Intrahepatic cholestasis of pregnancy (ICP), characterized by pruritus and elevated bile acid levels, affects approximately 0.5–5.0% of pregnancies and is the most common pregnancy-specific liver disorder [1,2,3]. The disease typically develops during the third trimester and is associated with adverse perinatal outcomes, including spontaneous preterm birth, fetal distress, meconium-stained amniotic fluid, and stillbirth, with risk increasing at higher bile acid concentrations [3,4,5]. Despite extensive research, the pathophysiology of ICP remains incompletely understood and appears to involve a complex interaction of genetic susceptibility, hormonal influences, environmental factors, and inflammatory mechanisms [2,5,6].

Recent evidence suggests that inflammatory and oxidative stress pathways may contribute significantly to the development and progression of ICP [2,7,8,9,10]. Elevated bile acid levels have been shown to activate inflammatory signaling pathways and promote placental dysfunction, supporting the hypothesis that immune and inflammatory responses play a role in disease pathogenesis [8,9,10]. However, clinically relevant biomarkers reflecting these inflammatory mechanisms are still limited.

Silent information regulator-2 protein 1 (SIRT1), also known as sirtuin-1, is a nicotinamide adenine dinucleotide (NAD+)-dependent deacetylase involved in regulating oxidative stress, inflammation, apoptosis, and cellular survival [11,12,13]. SIRT1 exerts anti-inflammatory and antioxidative effects by modulating several signaling pathways, including nuclear factor kappa-B (NF-κB), and plays an important role in vascular endothelial and placental function [14,15]. Additionally, SIRT1 is essential for normal trophoblast differentiation and placental development [15,16]. Experimental and clinical studies have shown that impaired SIRT1 expression is associated with abnormal placentation and adverse obstetric conditions such as preeclampsia, fetal growth restriction, and gestational diabetes mellitus [17,18,19,20].

Given the proposed inflammatory background of ICP and the anti-inflammatory role of SIRT1, altered maternal serum SIRT1 concentrations may reflect the inflammatory milieu associated with the disease. However, the relationship between maternal serum SIRT1 levels and ICP has not yet been investigated.

The aim of this prospective case–control study was to evaluate maternal serum SIRT1 levels in pregnancies complicated by ICP and compare them with healthy pregnancies, as well as to investigate their potential association with clinical and obstetric outcomes.

## 2. Materials and Methods

### 2.1. Study Design and Patient Selection

This prospective case–control study was conducted at the high-volume tertiary referral perinatology clinic of Ankara Etlik City Hospital, Ankara, Türkiye, between September 2025 and February 2026, in accordance with the principles of the Declaration of Helsinki. Ethical approval was obtained from the institutional ethics committee (AEŞH-BADEK2-2025-221, approval date: 16 September 2025), and written informed consent was obtained from all participants before enrollment.

Pregnant women in the second or third trimester who presented with pruritus suspicious for intrahepatic cholestasis of pregnancy (ICP) underwent fasting serum bile acid (SBA) testing and biochemical evaluation. Because the control group consisted of asymptomatic healthy pregnant women without pruritus or clinical suspicion of ICP, fasting serum bile acid measurements were not routinely performed in these participants. ICP was diagnosed in patients with fasting SBA levels greater than 10 μmol/L, according to the 2020 Society for Maternal-Fetal Medicine (SMFM) guideline [21]. ICP severity was classified according to fasting serum bile acid concentrations at the time of diagnosis as mild (≤40 μmol/L), moderate (41–99 μmol/L), or severe (≥100 μmol/L). Maternal blood samples for SIRT1 analysis were obtained at the time of diagnosis and before initiation of ursodeoxycholic acid (UDCA) treatment. After diagnosis, all patients with ICP were managed with UDCA therapy according to institutional clinical practice. In accordance with institutional clinical practice and contemporary guideline recommendations, elective induction of labor was generally performed at 37 weeks of gestation in pregnancies complicated by ICP unless earlier delivery was clinically indicated [21]. Whereas control pregnancies received routine obstetric care and were managed according to standard clinical indications.

**Figure 1:**
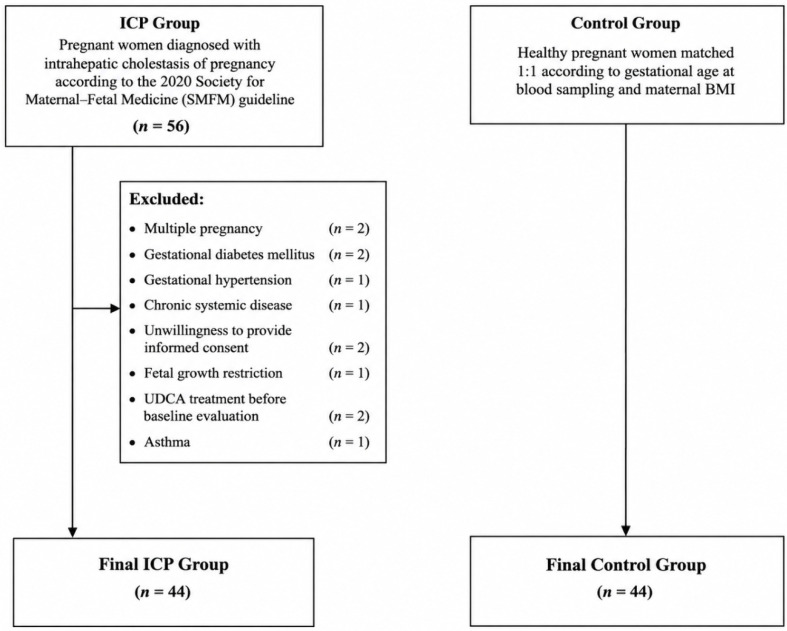
Flowchart of the study.

Exclusion criteria were multiple pregnancy (*n* = 2), gestational diabetes mellitus (*n* = 2), gestational hypertension (*n* = 1), chronic systemic disease (*n* = 1), unwillingness to provide informed consent (*n* = 2), fetal growth restriction (*n* = 1), initiation of UDCA treatment before baseline evaluation (*n* = 2), and asthma (*n* = 1). The control group included healthy pregnant women matched 1:1 with the ICP group by gestational age at blood sampling and maternal body mass index (BMI). In an additional analysis, small-for-gestational-age (SGA) was defined as birth weight below the 10th percentile for gestational age [22]. Clinical and laboratory data were obtained from the hospital electronic medical record system and patient files. A flowchart of patient selection is shown in Figure 1.

### 2.2. Sample Size Calculation

Since no previous clinical studies had evaluated maternal serum SIRT1 concentrations in pregnancies complicated by intrahepatic cholestasis of pregnancy, a reliable a priori sample size calculation could not be performed. Therefore, this study was designed as a prospective pilot case–control study intended to generate preliminary data for future larger investigations.

### 2.3. Biological Sample Collection and SIRT1 Analysis

Maternal serum samples for SIRT1 measurement were collected before the initiation of medical therapy and after an overnight fast of at least 8 h. Peripheral venous blood samples (2 mL) were obtained from all participants, allowed to clot at room temperature for 30–60 min, and centrifuged at 4000 rpm for 10 min. The separated serum samples were stored at −80 °C until biochemical analysis.

Maternal serum SIRT1 concentrations were measured using a commercially available enzyme-linked immunosorbent assay (ELISA) kit (Human SIRT1 ELISA Kit, Feiyuebio, Wuhan, China) according to the manufacturer’s instructions. Analyses were conducted in an independent biochemistry laboratory. The assay had a detection range of 0.32–20 ng/mL and an analytical sensitivity of 0.19 ng/mL. According to the manufacturer, both intra-assay and inter-assay coefficients of variation were less than 10%, and no significant cross-reactivity with analogous proteins was observed.

### 2.4. Statistical Analysis

Statistical analyses were conducted using the Statistical Package for the Social Sciences (SPSS) version 22.0 (IBM Corp., Armonk, NY, USA). The distribution of continuous variables was assessed with the Kolmogorov–Smirnov and Shapiro–Wilk tests. Normally distributed continuous variables are presented as mean ± standard deviation (SD), while non-normally distributed variables are reported as median and interquartile range (IQR).

Comparisons between two groups were performed using Student’s *t*-test or the Mann–Whitney U test, as appropriate. Categorical variables were compared using the chi-square test or Fisher’s exact test. Subgroup analyses within the ICP cohort were performed using one-way analysis of variance (ANOVA) or the Kruskal–Wallis test, according to data distribution.

Spearman correlation analysis was conducted to evaluate associations between maternal serum SIRT1 levels and clinical parameters. Receiver operating characteristic (ROC) curve analysis was used to assess the discriminative performance of maternal serum SIRT1 levels and combined predictive models for ICP.

Univariate and multivariable logistic regression analyses were conducted to investigate factors associated with ICP. Variables that were statistically significant in the univariate analysis were included in the multivariable model. Multicollinearity among variables in the multivariable analysis was assessed using variance inflation factor (VIF) values. Because of strong biological and statistical collinearity between AST and ALT, only ALT was included in the multivariable model. Model calibration was evaluated using the Hosmer–Lemeshow goodness-of-fit test, while overall model performance was assessed using omnibus model coefficients and Nagelkerke R^2^ values. A two-sided *p*-value less than 0.05 was considered statistically significant.

## 3. Results

A total of 88 pregnant women were included in the study: 44 women with ICP and 44 healthy controls. Demographic, clinical, and laboratory characteristics of the study population are summarized in Table 1.

Maternal age, gravidity, parity, number of abortions, body mass index (BMI), smoking status, and gestational age at blood sampling were similar between the groups (*p* > 0.05 for all). Serum alanine aminotransferase (ALT), aspartate aminotransferase (AST), uric acid levels, and platelet counts were significantly higher in the ICP group compared with controls (*p* < 0.05 for all), whereas serum albumin levels were significantly lower in women with ICP (*p* < 0.001). No significant differences were observed in blood urea nitrogen (BUN), activated partial thromboplastin time (aPTT), prothrombin time (PT), hemoglobin, hematocrit, leukocyte, lymphocyte, neutrophil, or monocyte counts between the groups. Maternal serum SIRT1 levels were significantly lower in the ICP group than in controls [1.06 (1.05) ng/mL vs. 1.54 (1.74) ng/mL, respectively; *p* = 0.005].

Obstetric and neonatal outcomes of the study groups are summarized in Table 2. Gestational age at delivery was significantly lower in the ICP group compared with controls [36.3 (1.0) weeks vs. 39.0 (3.0) weeks, *p* < 0.001]. Birth weight was also significantly lower in pregnancies complicated by ICP (2845 ± 587 g vs. 3284 ± 458 g, *p* = 0.001). Although birth weight was significantly lower in the ICP group, the frequency of SGA neonates (birth weight <10th percentile) was similar between groups (6.8% vs. 4.7%, *p* = 1.000), suggesting that the observed difference in birth weight was primarily related to earlier gestational age at delivery.

There were no significant differences between the groups in cesarean delivery rates, 1 min and 5 min Apgar scores, or neonatal intensive care unit (NICU) admission rates (*p* > 0.05 for all). No cases of stillbirth or neonatal death occurred in either group during follow-up.

Among women with ICP, 31 (70.5%) were classified as having mild disease, 9 (20.5%) as moderate disease, and 4 (9.0%) as severe disease according to fasting serum bile acid levels. Median fasting SBA levels were 19.50 (13.8) μmol/L in the mild ICP subgroup, 45.30 (16.7) μmol/L in the moderate subgroup, and 120.0 (79.3) μmol/L in the severe subgroup.

No statistically significant differences were demonstrated among ICP severity subgroups in age, ALT, AST, albumin, uric acid, BUN, aPTT, PT, hemoglobin, hematocrit, leukocyte, lymphocyte, neutrophil, or monocyte counts (*p* > 0.05 for all). Median maternal serum SIRT1 levels were 1.09 (0.93) ng/mL in the mild ICP subgroup, 0.80 (1.02) ng/mL in the moderate subgroup, and 1.27 (2.36) ng/mL in the severe subgroup, although no statistically significant difference was demonstrated between severity groups (*p* = 0.429); however, these findings should be interpreted cautiously.

Correlation analysis between maternal serum SIRT1 levels and clinical parameters is presented in Table 3. Among women with ICP, maternal serum SIRT1 concentrations were not significantly correlated with serum bile acid levels, ALT, AST, albumin, platelet count, or uric acid levels (all *p* > 0.05).

**Figure 2:**
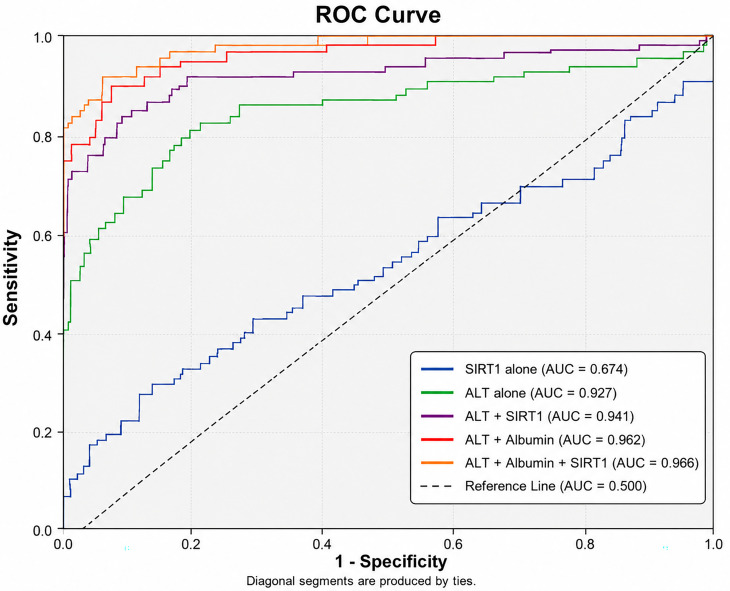
Receiver operating characteristic (ROC) curves of maternal serum SIRT1 alone and combined biochemical models for predicting intrahepatic cholestasis of pregnancy (ICP).

Receiver operating characteristic (ROC) curve analysis demonstrated modest discriminative performance of maternal serum SIRT1 alone for identifying ICP, with an area under the curve (AUC) of 0.674 (95% confidence interval [CI]: 0.559–0.788, *p* = 0.005). A SIRT1 cut-off value of ≤1.28 ng/mL yielded 63.6% sensitivity, 60.5% specificity, a positive likelihood ratio (+LR) of 1.61, and a negative likelihood ratio (−LR) of 0.60 (Table 4). In contrast, ALT alone showed excellent discriminative performance between the study groups (AUC: 0.927, 95% CI: 0.860–0.995, *p* < 0.001). However, because of the case–control design and the use of healthy controls, these findings should not be interpreted as estimates of real-world diagnostic accuracy. Combined ROC analyses yielded AUC values of 0.962 (95% CI: 0.925–0.999, *p* < 0.001) for the ALT + albumin model and 0.966 (95% CI: 0.933–0.998, *p* < 0.001) for the ALT + albumin + SIRT1 model. Because formal statistical comparison of these correlated ROC curves was not performed, the observed difference should be interpreted descriptively (Figure 2).

The overall multivariable logistic regression model including ALT, albumin, and maternal serum SIRT1 was statistically significant according to the omnibus test of model coefficients (χ^2^ = 83.912, *p* < 0.001). The model demonstrated acceptable calibration based on the Hosmer–Lemeshow goodness-of-fit test (χ^2^ = 2.860, *p* = 0.943). In addition, the model demonstrated a Nagelkerke R^2^ value of 0.825. This result should be interpreted cautiously because of the case–control design and limited sample size.

Exploratory univariable and multivariable logistic regression analyses were conducted to evaluate factors independently associated with ICP (Table 5). Increased ALT levels were independently associated with ICP, while higher albumin levels were inversely associated with ICP. Although platelet counts were significant in univariable analysis, this association did not remain after multivariable adjustment. Maternal serum SIRT1 levels were not independently associated with ICP in multivariable analysis.

## 4. Discussion

To the best of our knowledge, this is the first study in the English-language literature to investigate maternal serum SIRT1 levels in pregnancies complicated by intrahepatic cholestasis of pregnancy (ICP). Maternal serum SIRT1 concentrations were significantly lower in women with ICP compared with healthy pregnant controls. Although maternal serum SIRT1 alone demonstrated only modest discriminative performance and was not independently associated with ICP after adjustment for laboratory parameters, conventional liver function markers, particularly ALT and albumin, remained the primary contributors to diagnostic discrimination. These findings suggest that maternal serum SIRT1 may be more relevant as a marker of disease-related inflammatory and oxidative stress pathways than as a clinically useful diagnostic biomarker. Furthermore, the modest discriminative performance of SIRT1 and its minimal incremental contribution beyond conventional biochemical markers limit its potential applicability in routine clinical practice. In addition, the excellent performance observed in the combined ALT–albumin–SIRT1 model should be interpreted cautiously. Given the relatively small sample size and the absence of internal or external validation procedures, the possibility of model overfitting cannot be excluded. Therefore, these findings should be considered exploratory and require confirmation in larger independent cohorts before any clinical application can be considered.

In addition to biochemical differences, birth weight was significantly lower in the ICP group. However, this finding should be interpreted cautiously because pregnancies complicated by ICP were delivered significantly earlier, largely reflecting institutional management practices. Consistent with this interpretation, SGA rates were comparable between groups (6.8% vs. 4.7%, *p* = 1.000), suggesting that the observed difference in birth weight may primarily reflect gestational age at delivery rather than impaired fetal growth.

The pathogenesis of ICP is complex, and increasing evidence indicates that inflammatory pathways contribute to disease development [3]. Maternal inflammatory indices derived from peripheral blood samples further support this association [23,24,25]. Elevated maternal serum bile acid (SBA) levels may disrupt bile acid homeostasis and trigger inflammatory responses [3,10,26,27]. Additionally, reversal of bile acid transport at the fetomaternal interface may increase fetal bile acid exposure and contribute to adverse perinatal outcomes [27]. Experimental studies have shown that bile acids can activate the NF-κB signaling pathway, leading to upregulation of inflammatory genes in trophoblasts and abnormal placental inflammatory responses [8]. Morphological placental abnormalities have also been observed in placental tissue exposed to bile acids from cholestatic pregnancies [28].

SIRT1 is expressed in syncytiotrophoblasts, cytotrophoblasts, amniotic epithelium, chorion, and decidual cells, and is known to exert anti-inflammatory and antioxidative effects within placental tissues [29]. Experimental studies, including those using SIRT1 knockout mouse placentas, have shown that sirtuins play an important role in trophoblast differentiation and placental development [15,29,30]. Additionally, previous prospective clinical studies have reported reduced maternal and placental SIRT1 expression in obstetric disorders associated with placental dysfunction, such as preeclampsia and fetal growth restriction [17,18].

In this study, maternal serum SIRT1 levels were significantly reduced in pregnancies complicated by ICP. These findings further support the potential involvement of inflammatory and oxidative stress-related pathways in ICP pathophysiology and suggest that decreased maternal SIRT1 levels may reflect impaired anti-inflammatory regulation associated with the disease process.

Previous studies have investigated the role of SIRT1 in regulating oxidative stress and inflammatory pathways in various tissues. Singh et al. demonstrated that SIRT1 suppresses the transcriptional activity of nuclear factor kappa-B (NF-κB) in the liver and that reduced SIRT1 activity is associated with increased reactive oxygen species (ROS) production, hepatic inflammation, and non-alcoholic steatohepatitis [31]. Additionally, SIRT1 has been shown to regulate pro-inflammatory signaling pathways by suppressing tumor necrosis factor-alpha (TNF-α)-induced inflammatory responses and inhibiting activation of the IκB kinase (IKK)-NF-κB pathway [31]. Experimental studies have also highlighted the role of SIRT1 in hepatic lipid metabolism and the maintenance of metabolic homeostasis [32].

Although the precise origin of circulating SIRT1 could not be determined in this study, the observed reduction may reflect altered inflammatory or metabolic regulation involving both placental and hepatic tissues.

Interestingly, maternal serum SIRT1 concentrations were not significantly correlated with serum bile acid levels or other routinely assessed biochemical parameters among women with ICP. However, these findings should be interpreted with caution because the number of patients in the moderate and severe ICP subgroups was limited, particularly in the severe group (*n* = 4), reducing the statistical power to detect potentially meaningful differences according to disease severity. Therefore, the absence of a significant association should not be interpreted as evidence of no relationship between SIRT1 concentrations and ICP severity. Nevertheless, this finding may suggest that reduced SIRT1 levels reflect broader inflammatory, oxidative stress-related, and metabolic dysregulation accompanying ICP rather than directly mirroring the biochemical severity of cholestasis. Because SIRT1 participates in multiple cellular pathways regulating inflammation, oxidative stress, cellular homeostasis, and metabolic function, circulating SIRT1 concentrations may represent a systemic response to disease rather than a direct consequence of elevated bile acid levels [13,14,15,31].

In addition, lower maternal serum albumin levels were independently associated with ICP in multivariable analysis, possibly reflecting hepatic and inflammatory alterations accompanying cholestatic disease processes in pregnancy [2,7,10].

Previous studies have suggested that SIRT1-related pathways may have therapeutic relevance in placental and metabolic disorders. In a prospective study evaluating placental tissue from pregnancies complicated by fetal growth restriction, reduced SIRT1 expression was observed, and the authors proposed that SIRT1-related pathways could be potential therapeutic targets [18]. Experimental studies have also highlighted possible metabolic benefits of SIRT1 activation in obesity-associated metabolic disorders [32]. Although our findings do not establish a causal relationship, they support the possible involvement of SIRT1-related inflammatory and metabolic pathways in ICP pathophysiology. Further experimental and translational studies are needed to clarify whether modulation of SIRT1 activity may have therapeutic relevance in ICP.

Our findings are also consistent with a broader challenge in obstetric biomarker research. Several maternal serum biomarkers have demonstrated statistically significant associations with adverse pregnancy outcomes, yet their standalone predictive or diagnostic performance often remains modest. As highlighted in previous studies, statistically significant biological associations do not necessarily translate into clinically useful prediction tools, particularly when evaluated as isolated biomarkers [33]. Therefore, although reduced maternal serum SIRT1 concentrations may provide insight into the biological mechanisms underlying ICP, its limited discriminative performance suggests that SIRT1 should not be considered a clinically useful standalone diagnostic marker in its current form and may be better interpreted within a broader pathophysiological framework.

To the best of our knowledge, this is the first study in the English-language literature to investigate maternal serum SIRT1 levels in pregnancies complicated by intrahepatic cholestasis of pregnancy, providing new insight into the potential inflammatory and oxidative stress-related mechanisms involved in disease pathophysiology. The prospective study design and inclusion of a healthy control group matched for gestational age and BMI strengthen the reliability of the comparisons.

However, several limitations should be noted. First, this was a single-center study conducted in a tertiary referral hospital, which may limit the generalizability of the findings. Second, the relatively small sample size, particularly in the moderate and severe ICP subgroups, may have limited the statistical power of subgroup analyses. Third, although gravidity and parity were not significantly different between the groups, both variables demonstrated borderline statistical significance and may therefore represent potential residual confounding factors. Consequently, a modest influence of reproductive history on maternal serum SIRT1 concentrations and the observed associations cannot be completely excluded. Fourth, only maternal serum SIRT1 levels were evaluated; placental tissue expression and longitudinal SIRT1 measurements during pregnancy were not assessed. Furthermore, serum bile acid concentrations were not available in the healthy control group because bile acid testing was not clinically indicated in asymptomatic pregnancies. Finally, due to the observational nature of the study, causal relationships between reduced SIRT1 levels and ICP pathophysiology cannot be established.

In addition, the control group consisted of healthy pregnant women rather than symptomatic pregnant women with pruritus and normal bile acid concentrations. Therefore, the present study was designed primarily to investigate the biological association between ICP and maternal serum SIRT1 levels rather than to evaluate the real-world diagnostic performance of SIRT1. Future studies including clinically relevant comparison groups are needed to better define the potential diagnostic utility of SIRT1 in pregnancies presenting with pruritus.

## 5. Conclusions

In conclusion, although maternal serum SIRT1 levels were significantly reduced in pregnancies complicated by ICP, their diagnostic performance was modest and provided minimal incremental value beyond conventional biochemical markers such as ALT and albumin. Therefore, SIRT1 is unlikely to have substantial clinical utility as a standalone diagnostic biomarker for ICP. Nevertheless, the observed reduction in maternal serum SIRT1 levels supports the possible involvement of SIRT1-related inflammatory and oxidative stress pathways in ICP pathophysiology. Further mechanistic, translational, and placental tissue studies are warranted to better elucidate the biological role and potential clinical relevance of SIRT1 in ICP.

## Figures and Tables

**Table 1:** Demographic, clinical, and laboratory characteristics of the study groups.

Parameter	ICP Group (*n* = 44)	Control Group (*n* = 44)	*p* Value
**Maternal Characteristics**			
Age (years)	29.05 ± 4.8	28.77 ± 5.4	0.931
Gravidity (*n*)	2 (1)	2 (0)	0.058
Parity (*n*)	0 (1)	1 (1)	0.053
Abortus (*n*)	0 (0)	0 (0)	0.435
BMI (kg/m^2^)	28.0 (7.0)	29.2 (4.4)	0.584
Smoking status (*n*, %)	2 (4.5%)	0 (0%)	0.494
Gestational age at sampling (weeks)	33.45 ± 3.46	32.84 ± 3.50	0.411
**Liver and Biochemical Parameters**			
ALT (IU/L)	126 (226)	11 (7)	*<0.001*
AST (IU/L)	63.50 (101.0)	16.0 (8.0)	*<0.001*
Albumin (g/L)	34.65 (3.5)	37.00 (2.3)	*<0.001*
Uric Acid (mg/dL)	3.90 (1.3)	3.50 (1.1)	*0.002*
BUN (mg/dL)	14.15 (8.3)	15.10 (6.5)	0.623
**Hematological Parameters**			
aPTT (s)	27.16 ± 2.93	26.20 ± 2.45	0.101
PT (s)	8.32 (0.64)	8.31 (0.68)	0.721
Hemoglobin (g/dL)	11.53 ± 0.94	11.58 ± 1.22	0.918
Hematocrit (%)	35.97 ± 2.67	35.56 ± 3.18	0.374
Leukocyte count (×10^3^/mm^3^)	9.5 ± 2.45	10.43 ± 2.72	0.131
Lymphocyte count (×10^3^/mm^3^)	1.91 ± 0.55	1.93 ± 0.64	0.988
Neutrophil count (×10^3^/mm^3^)	6.68 (2.99)	7.64 (2.78)	0.122
Monocyte count (×10^3^/mm^3^)	0.65 (0.32)	0.65 (0.33)	0.596
Platelet count (×10^3^/mm^3^)	266.46 ± 67.31	235.86 ± 52.40	*0.008*
**Biomarker Analysis**			
SIRT1 (ng/mL)	1.06 (1.05)	1.54 (1.74)	*0.005*

Data are presented as mean ± standard deviation or median (interquartile range), according to data distribution. BMI: body mass index; ALT: alanine aminotransferase; AST: aspartate aminotransferase; BUN: blood urea nitrogen; aPTT: activated partial thromboplastin time; PT: prothrombin time; SIRT1: silent information regulator-2 protein 1. Significant values are italicized.

**Table 2:** Obstetric and neonatal outcomes of the study groups.

Parameter	ICP Group (*n* = 44)	Control Group (*n* = 44)	*p* Value
Gestational age at delivery (weeks)	36.3 (1.0)	39.0 (3.0)	*<0.001*
Cesarean delivery (*n*, %)	21 (47.7%)	23 (52.3%)	0.817
Birth weight (g)	2845 ± 587	3284 ± 458	*0.001*
Small for gestational age (SGA) (*n*, %)	3 (6.8%)	2 (4.7%)	1.000
1 min Apgar score	9.0 (1.0)	9.0 (1.0)	0.102
5 min Apgar score	10.0 (1.0)	10.0 (1.0)	0.161
NICU admission (*n*, %)	1 (2.27%)	1 (2.27%)	1.000

Data are presented as mean ± standard deviation or median (interquartile range), according to data distribution. NICU: neonatal intensive care unit, SGA: small for gestational age; defined as birth weight below the 10th percentile for gestational age. Significant values are italicized.

**Table 3:** Correlation analysis between maternal serum SIRT1 levels and laboratory parameters in women with ICP.

Parameter	r	*p* Value
ALT	0.044	0.775
AST	0.070	0.654
Serum bile acid	−0.131	0.396
Albumin	0.078	0.614
Uric acid	−0.013	0.932
Platelet count	−0.199	0.196

Spearman correlation analysis was used. ALT: alanine aminotransferase; AST: aspartate aminotransferase.

**Table 4:** Receiver Operating Characteristic (ROC) Analysis of Maternal Serum SIRT1 for the Prediction of Intrahepatic Cholestasis of Pregnancy.

Biomarker	LR+	LR−	Cut-Off *	Sensitivity	Specificity	AUC	95% CI	*p*-Value
**SIRT1**	1.61	0.60	≤1.28	63.6%	60.5%	0.674	0.559–0.788	*0.005*

* Cut-off values were determined according to the Youden index. Abbreviations: ROC: receiver operating characteristic; AUC: area under the curve; CI: confidence interval; LR+: positive likelihood ratio; LR−: negative likelihood ratio; SIRT1: silent information regulator-2 protein 1. Significant values are italicized.

**Table 5:** Univariable and multivariable logistic regression analyses for factors associated with ICP.

		Univariable			Multivariable	
**Parameter**	**OR**	**95% CI**	***p***	**aOR**	**95% CI**	***p***
ALT	1.147	1.067–1.233	*<0.001*	1.143	1.060–1.232	*<0.001*
Albumin	0.735	0.611–0.884	*0.001*	0.651	0.445–0.954	*0.028*
Platelet count	1.010	1.002–1.018	*0.013*	0.995	0.976–1.014	0.578
SIRT1	0.739	0.494–1.107	0.143	0.720	0.297–1.748	0.468

Data are presented as odds ratio (OR) and 95% confidence interval (CI). Variables with *p* < 0.05 in univariable analysis were included in the multivariable model. Multicollinearity was assessed using variance inflation factor (VIF) analysis. Significant values are italicized.

## Data Availability

The datasets used and/or analysed during the current study are available from the corresponding author upon reasonable request.
